# Identification of Bacterial Strains and Development of anmRNA-Based Vaccine to Combat Antibiotic Resistance in *Staphylococcus aureus* via In Vitro and In Silico Approaches

**DOI:** 10.3390/biomedicines11041039

**Published:** 2023-03-28

**Authors:** Muhammad Naveed, Muhammad Waseem, Tariq Aziz, Jawad ul Hassan, Syeda Izma Makhdoom, Urooj Ali, Metab Alharbi, Abdulrahman Alsahammari

**Affiliations:** 1 Department of Biotechnology, Faculty of Science and Technology, University of Central Punjab, Lahore 54590, Pakistan; 2 Department of Agriculture, University of Ioannina, 47100 Arta, Greece; 3 Department of Biotechnology, Quaid-e-Azam University, Islamabad 15320, Pakistan; 4 Department of Pharmacology and Toxicology, College of Pharmacy, King Saud University, Riyadh 11451, Saudi Arabia

**Keywords:** antibiotic resistance, *Staphylococcus aureus*, virulent genes, chimera protein, computational vaccine design

## Abstract

The emergence of antibiotic-resistant microorganisms is a significant concern in global health. Antibiotic resistance is attributed to various virulent factors and genetic elements. This study investigated the virulence factors of *Staphylococcus aureus* to create an mRNA-based vaccine that could help prevent antibiotic resistance. Distinct strains of the bacteria were selected for molecular identification of virulence genes, such as *spa*, *fmhA*, *lukD*, and *hla-D*, which were performed utilizing PCR techniques. DNA extraction from samples of *Staphylococcus aureus* was conducted using the Cetyl Trimethyl Ammonium Bromide (CTAB) method, which was confirmed and visualized using a gel doc; 16S rRNA was utilized to identify the bacterial strains, and primers of *spa*, *lukD*, *fmhA*, and *hla-D* genes were employed to identify the specific genes. Sequencing was carried out at Applied Bioscience International (ABI) in Malaysia. Phylogenetic analysis and alignment of the strains were subsequently constructed. We also performed an in silico analysis of the *spa*, *fmhA*, *lukD*, and *hla-D* genes to generate an antigen-specific vaccine. The virulence genes were translated into proteins, and a chimera was created using various linkers. The mRNA vaccine candidate was produced utilizing 18 epitopes, linkers, and an adjuvant, known as RpfE, to target the immune system. Testing determined that this design covered 90% of the population conservancy. An in silico immunological vaccine simulation was conducted to verify the hypothesis, including validating and predicting secondary and tertiary structures and molecular dynamics simulations to evaluate the vaccine’s long-term viability. This vaccine design may be further evaluated through in vivo and in vitro testing to assess its efficacy.

## 1. Introduction

*Staphylococcus aureus*, a Gram-positive, cocci-shaped, and immotile bacterium of the Firmicutes family, is associated with a wide range of infections in mammalian skin, various mucosal membranes, and the nostrils. This human pathogen is a significant source of infections contracted in the community or hospital, with antibiotic resistance making it difficult to treat these infections effectively [1]. Due to its metabolic adaptability and pharmacy resistance, *Staphylococcus aureus* can thrive in a wide range of conditions, with an estimated 25–30% of healthy individuals harboring the bacteria on their skin and nasopharyngeal membranes as a natural component of the human microbiome (Raafat et al., 2020). *Staphylococcus aureus* is responsible for tens of thousands of infections annually and is a leading cause of pneumonia and some respiratory tract infections, cardiovascular infections, nosocomial infections, and prosthetic joints. Studies have shown that more deaths occur because of *Staphylococcus aureus* bacteremia than from AIDS, viral hepatitis, tuberculosis, and other diseases combined [2]. Furthermore, this bacterium is also associated with abscesses, wound infections, and furuncles, which can cause substantial morbidity and sickness but are rarely life-threatening.

However, when *Staphylococcus aureus* enters the circulation or internal tissues, it causes a wide range of severe illnesses. The emergence of antibiotic-resistant strains of bacteria, particularly methicillin-resistant *Staphylococcus aureus* (MRSA), has become a major public health issue due to the wide range of diseases it can cause, from mild skin infections to life-threatening conditions [3]. *Staphylococcus aureus* infections are typically treated with antibiotics as the primary means of defense. However, the impulsive use of antibiotics, which initially had a high success rate, led to the emergence and expansion of antibiotic-resistant bacteria. For instance, MRSA and vancomycin-resistant *staphylococcus aureus* (VRSA) were both discovered within a short period after the clinical application of these antibiotics [4]). The low affinity of penicillin-binding protein 2a (PBP2a) encoded by the *mecA* gene of *Staphylococcus aureus* for β-lactam medications, combined with the high production of the lysyl-phosphatidylglycerol enzyme, has caused methicillin-resistant *Staphylococcus aureus* to be resistant to β-lactam antibiotics. Additionally, *Staphylococcus aureus* produces the antibiotic-inactivating enzyme β-lactamase and, without the auxiliary gene regulator (Agr), β-lactam antibiotics are rendered ineffective [5]).

*Staphylococcus aureus* infections can be particularly severe due to their limited virulence factors, making it difficult for them to be fought by the body’s immune system. These virulence factors, mostly found on the surface of the bacteria, include proteins such as protein A, clumping factor, and binding proteins, as well as polysaccharide intercellular adhesins and toxins that function as superantigens. Enzymes, including coagulase, staphylokinase, and protease, aid in immune evasion and host tissue penetration. The production of *Staphylococcal* protein A and surface proteins by virtually all *Staphylococcus aureus* isolates serves as a superantigen by binding to immunoglobulins, inhibiting opsonization, and phagocytosis [6]. Adhesion and evasion of the immune response depend on clumping factors A and B, which bind to fibrinogen facilitating the invasion of host tissues. These proteins can also adhere to the extracellular matrix, promoting the invasion of host tissues through binding to fibronectin and elastin [7].

*Staphylococcus aureus* releases toxins and virulent genes associated with toxic shock syndrome, food poisoning, *Staphylococcal* scalded skin syndrome, and *Staphylococcal* scarlet fever. Moreover, these genes encode diverse virulent factors that play a crucial role in disease pathology. Factors such as toxic shock syndrome toxin-1, delta-hemolysin (*hld*), and *Staphylococcal* protein A (*Spa*) are all involved in the pathogenesis of bacterial infections. To combat the prevalent multidrug resistance of *Staphylococcus aureus*, this study aimed to identify virulent factors of the bacterium and construct an mRNA vaccine utilizing an immunoinformatic approach and various online bioinformatics tools. Additional tests utilizing both in vitro and in vivo studies on mRNA vaccines will be required to assess the efficacy of an mRNA vaccine in the future.

## 2. Materials and Methods

### 2.1. Sample Acquisition

Samples of various strains of *Staphylococcus aureus* were obtained from the Sheikh Zaid Hospital in Lahore and the Pakistan Council of Scientific and Industrial Research (PCSIR) laboratory in Lahore. The majority of specimens obtained were either blood or pus specimens, which were then refrigerated at −4 °C in the Molecular Biotechnology and Bioinformatics Laboratory of the University of Central Punjab in Lahore to be analyzed further.

### 2.2. Culture of Strains

Luria Bertani (LB) media was prepared in flasks using a specific chemical composition and was sterilized in an autoclave along with the petri plates that would be used during the experiment. After sterilization, the LB media was placed in a biosafety cabinet. The plates were cooled for 24 h before inoculation with the sample. After inoculation, the plates were incubated at 30 °C for better growth. Colonies were extracted from the bacterial cultures using a sterile loop and then streaked onto new plates for further analyses.

#### 2.2.1. DNA Isolation and 16s rRNA Characterization

The CTAB (DNA-kit) (Thermo Fisher Scientific, Waltham. MA, USA) method was employed to extract DNA from bacterial isolates according to manufacturer instructions, which were then subjected to PCR testing for complete molecular characterization of the samples. DNA was confirmed using agarose gel electrophoresis. The 16S rRNA gene was amplified by universal primers (27F/1492R) in a thermocycler. The polymerase chain reaction (PCR) is a thermocycler-based method that operates on the temperature gradient under optimal reaction conditions. The 16S rRNA is a universal sequence that is found in all bacteria but with some minor variations. Amplification of 16S ribosomal RNA was used to distinguish between different strains of bacteria. Primers 9F and 1510R were utilized for the purpose of amplification and identification of unidentified bacterial strains. In autoclaved PCR tubes, the PCR reaction was prepared by adding master mix, forward and reverse primer, DNA, and double distilled water profile of 16S rRNA. After preparing the reaction mixture (Table 1), the PCR profile mentioned in Table 2 was selected and the PCR cycles were adjusted to 35−38 cycles.

#### 2.2.2. Gel Electrophoresis Procedure

The solidified gel was placed in case trays and then inserted into gel electrophoresis containing TAE buffer. To detect DNA, a 6X loading dye was added, which resulted in clear bands. A mixture of 2 μL of 6X loading dye and 3 μL of DNA PCR sample was poured into the well, with the first well containing a ladder and the others containing DNA samples. The gel electrophoresis was covered with lead and a voltage was applied to separate the bands based on charge-to-mass ratio; the recommended voltage range was between 85–100 V. After the separation process was complete, the gel was transferred from the electrophoresis apparatus to a gel documentation system using gloves. The bands were visualized using UV light.

#### 2.2.3. Strain Identification

The Ez-Taxon service, based on similarities between sequences and databases of 16S rRNA sequences, was used to identify strains. The Ez-Taxon service analyzed the 16S rRNA sequences to identify any previously unidentified strains [8]. The BLAST search tool (https://blast.ncbi.nlm.nih.gov/Blast.cgi, assessed on 18 January 2023) [9], an online search engine that compares provided nucleotide sequences of *Staphylococcus aureus* strain MBBL19 which has accession number ON875315 with other sequence databases, was used to identify strains based on homology (95.89%) and query coverage (97%).

#### 2.2.4. Amplification of *LukD, FmhA*, *Spa* and Delta Hemolysin

After identifying or confirming the bacterial strain through 16srRNA, the next step involved identifying various virulent or resistant genes. In different strains of Staphylococcus, the genes *LukD*, *FmhA*, *Spa*, and *Delta Hemolysin* were amplified, each under specific PCR conditions, provided in Table 2. Following amplification, the genes’ bands were visualized on a 2% agarose gel, confirming their presence in the various Staphylococcus strains. To confirm the band and gene sizes, the samples were run on an agarose gel with a 1Kb ladder after amplification was completed.

#### 2.2.5. Phylogenetic Analysis

A neighbor-joining technique was used to build a phylogenetic tree with the MEGA X software program. The ClustalW program within MEGA X (downloaded from https://www.megasoftware.net/, assessed on 20 January 2023 [10]) was used to align multiple homologous sequences assigned by BLAST and to group them into a single entity. A bootstrap value of 1000 was employed to ensure the consistency of the association.

### 2.3. Chimeric Structure Design

The sequencing revealed the presence of the *Staphylococcal* protein A (*spa*), Delta-hemolysin (*hld*), and *fmhA* gene sequences. The ClustalW program (http://www.ebi.ac.uk/Tools/clustalW2, assessed on 20 January 2023 [11]) from the European Bioinformatics Institute’s website was utilized to perform multiple alignments to identify consistent segments within all the gene sequences. To create a synthetic chimeric protein, genes were converted into proteins using the Expasy Protparam tool (https://web.expasy.org/protparam/, accessed on 23 January 2023 [10]) and hydrophobic linkers were utilized to join antigenic regions in these proteins.

### 2.4. Immune Cells Epitopes Prediction

The ABCpred web server was employed to predict B-cell’s linear epitopes (https://webs.iiitd.edu.in/raghava/abcpred/, assessed on 23 January 2023 [12]). The Immune Epitope Database (IEDB’s) MHC-II server was utilized to predict HLA epitopes by selecting proteins with a cutoff of 0.5. The sequence submitted to the server (http://tools.iedb.org/mhcii/, accessed on 25 January 2023) was formatted in FASTA (NCBI, Bethesda, MD, USA), and the parameters of NN-align 2.3 were used to anticipate the epitopes of the sequence [13]. These epitopes had a length of 15-mer, and those with an IC50 value of up to 100 were identified. CTL epitopes were predicted using the IEDB’s MHCI server (http://tools.iedb.org/processing/, ANN version 4.0, assessed on 25 January 2023 was) used to predict the epitopes of the chosen protein based on its sequence [14]. Potential epitopes were taken from the entire human HLA database. An IC50 value of less than 100 was applied to the vaccine candidate for epitope selection.

#### 2.4.1. Homology Analysis of Predicted Proteins

The NCBI BLASTp database (https://blast.ncbi.nlm.nih.gov/Blast.cgi, accessed on 26 January 2023) was used to look for likely peptides that could preclude the possibility of autoimmunity [15]. Any E values greater than 0.05 for the rest of the peptides were viewed as potential non-homologous peptides and used in the procedure for vaccine development.

#### 2.4.2. Antigenicity and Allergenicity Analysis of Epitopes

VaxiJen website (http://www.ddg-pharmfac.net/vaxijen/VaxiJen/VaxiJen.html, accessed on 26 January 2023) and AllerTOP version 2 (https://www.ddg-pharmfac.net/AllerTOP/, accessed on 27 January 2023) were used as per the technique narrated by [16]. A 0.5 threshold was used for the assessment of antigenicity and default parameters for the evaluation of allergenicity. Only epitopes that had been determined to be both antigenic and not allergenic were kept for vaccine development.

#### 2.4.3. Population Coverage Analysis

Population coverage was analyzed with the Population Coverage tool (http://tools.iedb.org/population/, accessed on 27 January 2023) from the IEDB database to estimate the immunization formulation’s applicability to specific T-cell epitopes and related MHCI and MHCII alleles based on the approach outlined by [17]. The range of MHC alleles present was identified by the construct’s epitopes, which were observed to vary across different regions and populations.

### 2.5. mRNA Vaccine Construct

**Figure 1:**
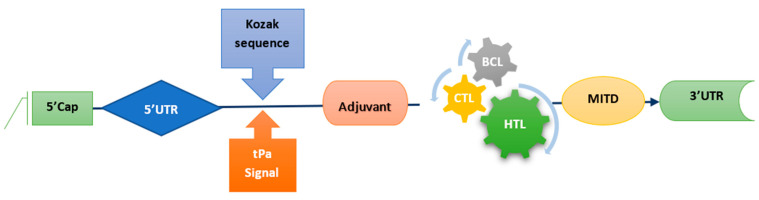
Specifics of the vaccine construct.

A synthetic mRNA vaccine construct was developed by linking the recommended epitopes using the linkers GPGPG, AAY, and KK, to allow for the independent function of epitopes. A RpfE booster was used to amplify the delayed reaction of immune system cells. The mRNA-based vaccine design also incorporated a Kozak sequence, as well as tPA (P00750) and MITD (Q8WV92) in the 5′ and 3′ regions, respectively, as per the methodology described by [18] and shown in Figure 1.

### 2.6. Prediction of Physicochemical Properties of Vaccine Construct

The capacity of the vaccine candidate to trigger an antigenic or allergic response was estimated by employing a combination of VaxiJen version 2.0 (http://www.ddg-pharmfac.net/vaxijen/VaxiJen/VaxiJen.html, accessed on 28 January 2023) and ANTIGENpro server (http://scratch.proteomics.ics.uci.edu/, accessed on 31 January 2023) [19]. The vaccine’s physiochemical features were utilized for antigenicity prediction by VaxiJen [19] while a machine-learning approach was employed by the ANTIGENpro server to identify results. The allergenicity of the construct was also estimated using the ToxinPred (http://crdd.osdd.net/raghava/toxinpred/, accessed on 1 February 2023 [20]) and AllerTOP servers (http://www.ddg-pharmfac.net/AllerTOP/, accessed 1 February 2023). The ProtParam program was utilized to ascertain the construct’s makeup, molecular weight, and predicted isoelectric point [21] and calculate the GRAVY score.

#### 2.6.1. Immune Simulation of Vaccine Construct

The C-Immsim, an internet-based program (http://150.146.2.1/CIMMSIM/index.php, assessed on 1 February 2023), was used to evaluate the immunological effects of the vaccine through simulation [22]. The simulation typically requires between two and three doses of vaccination that are administered over a period of four weeks. The default parameters employed in the simulation were two injections at hour one, three and a half days, and seven days.

#### 2.6.2. Structures Prediction and Validation of Vaccine Construct

Prediction and validation of the secondary and tertiary structures of the mRNA vaccine were accomplished using a combination of computational tools. The Vienna RNA Package 2.0′s RNAfold program from https://www.tbi.univie.ac.at/RNA/, assessed on 2 February 2023 [23]) was utilized to estimate the minimum free energy (MFE) of the secondary structure, employing the McCaskill algorithm. Moreover, to predict secondary and tertiary structures, the PSIPRED server (http://bioinf.cs.ucl.ac.uk/psipred/, assessed on 3 February 2023 [24]) and Robetta server (https://robetta.bakerlab.org/, assessed on 4 February 2023 [25]) respectively, were employed. The predicted structures were subsequently validated through a series of online servers, including PROCHECK, ERRAT, and Ramachandran Plot, available at https://saves.mbi.ucla.edu/ (assessed on 5 February 2023 [26]), and the ProSA-webserver (https://prosa.services.came.sbg.ac.at/prosa.php, assessed on 5 February 2023 [27])

### 2.7. Docking of Vaccine Construct with TLR-3 Receptor

The possible encounter between the vaccine structure and human Toll-like receptor 3 (TLR-3) was assessed through protein–protein docking with the ClusPro server https://cluspro.bu.edu/home.php [28]. The vaccine formulation was used as a ligand, and the 3D structure of TLR-3 (PDB ID 2A0Z; obtained from the Protein Data Bank (https://www.rcsb.org/, assessed on 6 February 2023 [29]) was utilized as the receptor. The resulting complexes were analyzed to determine the suitability of the interaction based on the energetics of the docking.

### 2.8. Molecular Dynamic Simulation

The resultant complexes were then submitted to molecular dynamics simulation analysis (http://www.imods.Chaconlab.org/, assessed on 7 February 2023) to confirm the atoms’ and molecules’ stability and mobility within the vaccine construct [30]. 

### 2.9. Computational Expression Studies

The process of optimizing the designed vaccine construct began with converting the amino acid sequence to a DNA sequence through the reverse translate tool and the universal codon table [31], available at https://www.bioinformatics.org/sms2/rev_trans.html, (assessed on 8 February 2023). Subsequently, the sequences of the DNA were modified for successful production in *Homo sapiens* [32]) using Jcat at http://www.jcat.de/, accessed on 8 February 2023. Lastly, the optimized sequence of DNA was computationally expressed in a pET28a (+) expression vector of 5369 bps using Snapgene, downloaded from https://www.snapgene.com/ (accessed on 8 February 2023) and following the methodology of [33]. This process resulted in the final optimized vaccine construct. The BgIII site was flanked at position 401, allowing the automatic filling of overhangs, and the PshAI site was flanked at 1968. The gap was replaced by the optimized DNA construct of 1215 bases.

## 3. Results

### 3.1. Sample Collection and Strain Identification

**Figure 2:**
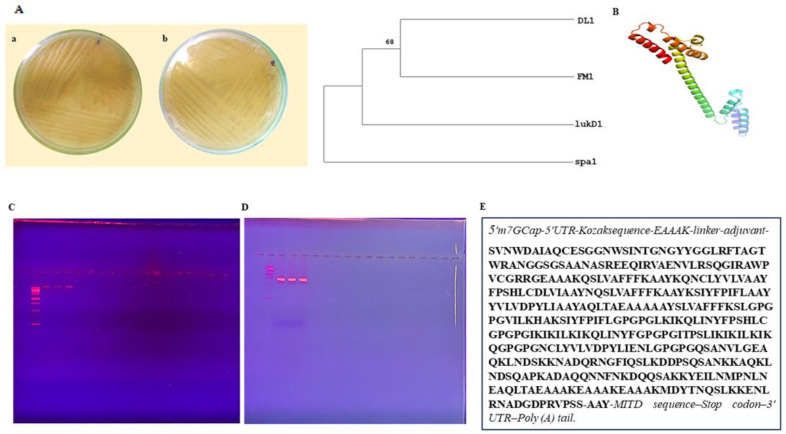
Vaccine design using chimeric protein derived from three Staphylococcal genes. (**A**). Colonies of *Staphylococcus aureus* on L.B agar plates (**a**) and (**b**) showing streaked culture plates of *Staphylococcus aureus* (**B**) Phylogenetic Analysis of Sequenced Virulence genes of *Staphylococcus aureus* using 1000 bootstrap value. Three virulence genes *spa*, *fmh*, and *hla-D*, were translated into proteins using Expasy Translator (SIB Swiss Institute of Bioinformatics, Lausanne, Switzerland), and the chimeric structure of these proteins was formed using linkers. (**C**) Illustration of DNA bands seen in the gel dock along with the ladder. (**D**) PCR Amplified 16S RNA seen in gel dock, confirming bacterial origin. (**E**) The final vaccine construct.

Samples were collected from Sheikh Zaid Hospital and the Pakistan Council of Scientific and Industrial Research (PCSIR) and placed on L.B. or nutritional agar. Colonies shown in panel 1 of Figure 2 were observed. DNA was extracted using the CTAB method and verified by gel electrophoresis alongside a 1 Kb ladder (Figure 2C), and 27F/1492R primer set with a 1 Kb ladder was used to amplify the isolated DNA for the 16S rRNA gene (as shown in Figure 2D). Applied Biosciences International (ABI) sequenced various bacterial strains and compared them to other strains through the Blast program. A phylogenetic tree was then constructed based on the sequenced strains, as shown in Figure 2A. The taxonomy of isolated bacterial species was identified using the Ez-Taxon website. According to the findings, all isolates were found to be from the *Staphylococcaceae* family.

### 3.2. Phylogenetic Analysis

The MEGA-X program constructed the phylogenetic trees using the maximum composite likelihood approach to determine the evolutionary distance in nucleotide substitutions per site. The phylogenetic tree was constructed using 1000 bootstrap values. The resulting datasets for ST1, ST2, and ST3 contain nucleotide sequences, respectively. Isolate phylogenetic trees are depicted in Figure 2A. The 16S rRNA sequences were edited to exclude the unrefined sequence.

### 3.3. Chimeric Design

For the construction of chimeric protein structure, sequenced genes were converted into respective proteins using Expasy ProtParam. The EAAAKEAAAKEAAAK repetitions and the DPRVPSS repeats were utilized to create linkers that separated the three domains and resulted in chimera formation, as shown in Figure 2B.

### 3.4. Immune Cells Prediction and Estimation

As indicated in Table 3, four of the top ABCpred-predicted epitopes were chosen from chimeric proteins. Only antigenic, nontoxic, non-allergenic, and non-homologous epitopes were selected to build vaccines. To ensure that vaccine design does not create autoimmunity, every isolated epitope was examined to see if it was comparable to *Homo sapiens*. The six B-cell epitopes that start the targeted proteins were evaluated for inclusion in our vaccine design (Table 3). CTL epitopes among the proteins were identified by using IEDB’s MHC-I database. The IC50 was approximately 100, and the ANN-predicted approach was used to pick epitopes in this study. Epitopes were screened for their ability to neutralize pathogens while being free of toxins or allergens. As a result, eight epitopes were selected for vaccine development, as shown in Table 4. To find possible HTL epitopes, proteins on the outer membrane were screened. Five HTL epitopes were screened for vaccine design, displayed in Table 5. Only antigenic, nontoxic, hypoallergenic, and ultimately non-homologous epitopes were chosen for this study because they could generate IL-4, INF-gamma, and Il-10 and generate a specific B-cell, HTL, and CTL response.

### 3.5. mRNA Vaccine Construct and Physiochemical Analysis

Several methods, including VaxiJen, ANTIGENpro, and AllerTOP, were utilized to assess the immunogenicity, allergy potential, toxicity, and solubility of the final construct shown in Figure 2E. The vaccination was confirmed to be antigenic, non-allergenic, nontoxic, and soluble. As shown in Table 6, the ExPasy ProtParam service was utilized to establish a construct’s physiochemical profile. The vaccine’s physiochemical properties predicted thermal stability. The vaccine was determined to be hydrophilic by the GRAVY value of −0.419. Based on these findings, the mRNA vaccine design might be a viable vaccination option for future clinical trials.

### 3.6. Predicted Population Coverage

MHC-I & II and the matching 14 epitopes were used to estimate worldwide coverage using the IEDB population coverage method. The predicted world vaccination rate would be approximately 90.57%.

### 3.7. Immune Simulation

**Figure 3:**
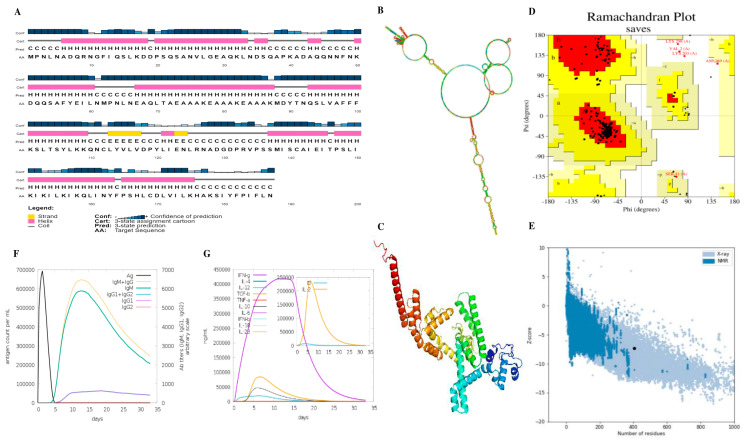
Physicochemical characteristics of the designed vaccine candidate and immune simulation predictions. (**A**) Prediction of Vaccine’s 2-D structure construct by PsiPred server; (**B**) Structure of stable mRNA with −948.30 kcal/mol and the energy of the centroid structure was −242.68 kcal/mol. (**C**) 3D structure anticipated using Robetta; (**D**) Ramachandran plot illustrated amino acid residues in different regions of vaccine construct; (**E**) ProSA web run for Z-s analysis; (**F**) Antibody production after vaccine inoculation as antigen; (**G**) Simulation of interferon, interleukins, and tumor necrosis factors.

Three vaccine doses were used to estimate the immune response. The second and third replies fared better than the first one. The immunoglobulin levels rose following antigen suppression, and it was discovered that more IgM was produced than IgG, as shown in Figure 3F. Immunological memory may have been established in response to antigen stimulation following the increase in this metric. The present isotypes of B-cells over a long period indicate that the B-cell population has formed memories. CTL and HTL cells proliferated in more significant numbers as memory developed. The activity of dendritic cells remained the same, but the activity of macrophages increased. IFN- and IL-2 concentrations also rose (Figure 3G). Immune system development was accompanied by increased innate immunity and epithelial cell numbers.

### 3.8. Structures of mRNA Vaccine Construct

According to the findings, the free energy of the mRNA vaccine was −948.30 kcal/mol, and the energy of the centroid structure was −242.68 kcal/mol kcal/mol. A stable mRNA structure has been demonstrated in Figure 3B. An analysis using the PSIPRED web tool demonstrated that the secondary structure of the vaccine, as depicted in Figure 3A, was composed predominantly of alpha helices. The vaccine’s tertiary structure was also deduced with the Robetta service, as shown in Figure 3C. PROCHECK was used to confirm the structure’s stereochemical accuracy; 94.8% of residues were mostly in desired zones, 3.8% were in the different allowed zones, and 0.3% were in the generously acceptable zones, according to the Ramachandran plot (depicted in Figure 3D). The vaccine has an overall quality factor of 99.49. A negative Z-score of −7.35 (Figure 3E) in the tertiary protein model was predicted by the ProSA-web server, which indicated that it is incredibly consistent.

### 3.9. Molecular Docking of Vaccine Construct with TLR-3

**Figure 4:**
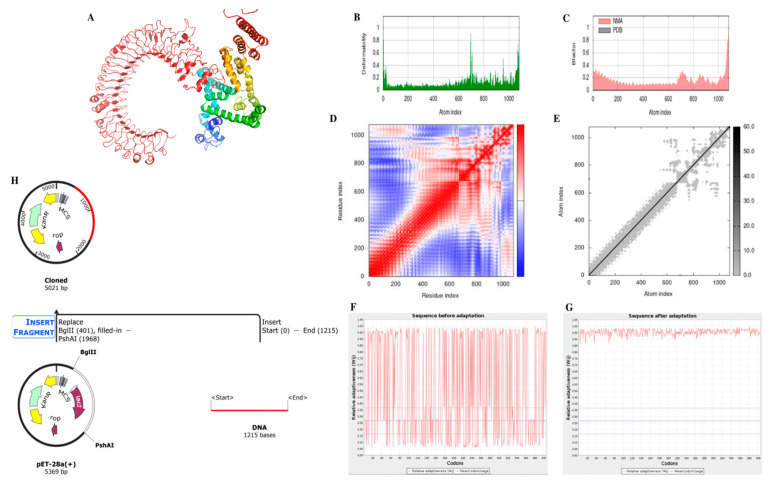
(**A**) Vaccine-receptor docking interaction using TLR-3 with red indicating the domain of receptor for ligand binding and the multicolor structure representing the vaccine candidate; (**B**) Graph for deformability; (**C**) Graph for B-factor; (**D**) A matrix of comparable fluctuation, showing alignment between couples of parts, for instance, whether they experience associated (red), unrelated (white), or opposing (blue) movements.; (**E**) A network of springs is depicted with a graphical model, where each pair of atoms connected by a spring is indicated. Every dot on the chart represents a single spring between two atoms, and the darkness of the shade shows the degree of stiffness of the spring, with darker grays indicating more substantial stiffness and lighter grays signifying weaker stiffness; (**F**) Sequence before optimization for expression in *Homo sapiens*; (**G**) Sequence after optimization for expression in *Homo sapiens*; (**H**) In silico cloning of the optimized DNA sequence in the pet 28A (+) expression vector.

The ClusPro software package accomplished molecular docking and validation of the construct’s interactions with TLR-3 receptors. The result from the ClusPro server showed ten possible docking models, and the best-fitting model was chosen. This model had the lowest energy score of −1255 and the lowest bond energy of −19.45 kcal/mol, as shown in Figure 4A.

### 3.10. Molecular Dynamic Simulations

The vaccine-TLR3 complex molecules were put through the iMODs server to perform the molecular dynamic simulation. A graph displaying the deformability of the vaccine (Figure 4B) illustrates how the various amino acids can coil up in response to increased spikes’ altitude levels. The flexibility of proteins was investigated utilizing NMA (normal mode analysis), which is a computer-based methodology. (Figure 4C). Figure 4D shows a covariance matrix that displays the correlations and anti-correlations between amino acid pairs in the vital area; the blue section reveals no associated residues. Similarly, the elastic network map in Figure 4E indicated the swift atomic interactions between the vaccine-receptor complex.

### 3.11. Computational Expression Studies

The reverse-translated DNA sequence was optimized for in silico cloning analysis. The sequence with an initial CAI score of 0.35 and 55.72% GC content (Figure 4F) was optimized to have a CAI value of 0.96 and a GC content of 66.42% (Figure 4G). A CAI-value of 1 represents the ideal expression indicating the efficacy of our construct optimization. As shown in Figure 4H, the BgIII site was flanked at position 401, and the PshAI site was flanked at 1968 to clone the DNA sequence.

## 4. Discussion

The bacterium *Staphylococcus aureus*, widely acknowledged for its ability to cause a spectrum of illnesses, particularly in individuals with nosocomial infections, has been reported to possess natural resistance to ampicillin, macrolide antibiotics, cephalosporins, and cefotaxime. The phenomenon of antibiotic defense in bacteria is a worldwide health issue, as it affects both animals and humans [34]. The increasing opposition to penicillin in *Staphylococcus aureus* has caused more frequent use of vancomycin to treat resistant bacterial infections worldwide. However, the frequent use of vancomycin has diminished its effectiveness in combating *Staphylococcus aureus* infections [35]. Considering these challenges, this study aims to identify virulent factors of *Staphylococcus aureus* to design an mRNA vaccine that can combat antibiotic resistance. The process involves isolating desired bacteria through culturing, selecting three strains for molecular identification, and detecting virulent genes, such as *spa*, *lukD*, *fmhA*, and *hld* genes, by means of PCR with the product size of 293 bp, 243 bp, 345 bp and 357 bp, respectively. DNA extraction from samples of *Staphylococcus aureus* obtained from the Sheikh Zayed Hospital and the PCSIR was performed utilizing the CTAB technique and confirmed by the gel doc technique. Identification of bacterial strains was accomplished by utilizing 16S rRNA and primers such as *spa*, *lukD*, *fmhA*, and *hld* for gene amplification. Sequencing was conducted at ABI, Malaysia, and the phylogenetic tree, and alignment of strains was constructed after sequencing. In silico analysis of *spa*, *fmhA*, and *hld* genes was also carried out, including creating an antigen-specific vaccine as performed by [36].

Vaccination aims to produce a sustained immune reaction in the individual, allowing the body to confront future encounters with the pathogen more effectively [37]. In this study, virulence genes were converted into proteins, and a chimeric construct was created using various linkers. Incorporating epitopes that can activate both B and T cells into the vaccine design is essential for the vaccine to be successful [38]. The chosen epitopes should be able to initiate the production of HTL-regulated anti-pathogen cytokines such as IFN-, IL-4, and IL-10 [39]. After an infection is cleared, only memory cells survive, while other immune cells die. Through the presentation of processed epitopes via MHC II, B cells recognize and deliver these epitopes to T cells, allowing the T-cell receptor (TCR) to acknowledge them.

Furthermore, B-cells develop into plasma cells that create antibodies and memory cells that can be called upon in the future [40]. This study emphasized the development of an mRNA peptide vaccine, created with antigenic proteins of *Staphylococcus aureus* and utilizing only in silico methods, that was designed to induce a robust immune response. The proposed mRNA vaccine’s antigenicity, non-allergenicity, and hydrophilicity were assessed using immunoinformatic techniques; the efficacy of these tools has been discussed in the work by [41,42,43]. Palladini et al. discussed that the predictions made by in silico modeling, which involves using computer simulations to predict outcomes, were accurate. However, designing and creating models of vaccines and protocols for vaccination should consider the gradual weakening of the immune system that occurs with aging and aim to enhance immune responses in older individuals [41]. To research a vaccine’s efficacy, this study used molecular simulations to determine if it induced an immune response following three injections. The simulations’ results showed that the response could generate memory cells and chemokines that stimulate B-cell and humoral reactions. The effective use of molecular dynamics simulations and the accuracy of computational tools has been confirmed previously [44,45,46]. Indicators such as macrophages and dendritic cells also showed signs of memory cell formation. Ultimately, this study determined that vaccination is viable for preventing *Staphylococcus aureus* infections.

The primary advantage of using in silico tools is that they allow for the rapid and efficient identification of virulent genes and the design of an mRNA vaccine. The design can be completed in relatively short time lapses and at a lower market price compared to traditional laboratory techniques. Additionally, in silico techniques can provide a high degree of accuracy and specificity in identifying virulent genes, which is crucial for the success of the vaccination. However, there are also limitations to using only computational tools. In silico techniques are dependent on the availability and quality of data, and the validity of predictions is highly reliant on the algorithms used.

Furthermore, the results of the in silico analysis must be verified experimentally to ensure their validity. The study suggests that vaccination is a viable option for preventing *Staphylococcus aureus* infections, and that in silico techniques can help identify virulent genes and design an mRNA vaccine. However, it is essential to consider the limitations of using only computational tools and to verify the results experimentally.

## 5. Conclusions

With the proliferation of antibiotic resistance among bacterial strains, identifying and targeting virulent factors are of utmost importance in developing effective therapeutics. This research employed a multi-disciplinary approach, utilizing in silico techniques, molecular biology, and immunological analysis to identify and target resistant and virulent genes of *Staphylococcus aureus*. The sequencing of these genes was then used to construct a chimeric protein structure that served as the basis for the model of an mRNA-based antibiotic. This vaccine was then validated through various in silico simulations, and its immunological properties were analyzed using immune-informatics techniques. The outcomes of this study demonstrate that the vaccine construct possesses advantageous physicochemical properties and elicits a robust immune response against *Staphylococcus aureus*. Further studies, including in vivo and in vitro trials, are required to fully assess the potency of this vaccine design in combatting infections caused by this virulent pathogen.

## Figures and Tables

**Table 1:** PCR reaction mixture preparation and composition.

Ingredient	Amount (μL)
Primer1 (Forward)	1
Primer2 (Reverse)	1
ddH2O	8.5
Master mix	12.5
Sample of DNA	2
Total Amount	25

**Table 2:** PCR profiles for strain and genes’ amplification.

Profile for 16S rRNA
Adjust the initial temperature to 95 °C for 5 min.
Adjust the time for denaturation to 30 s at 95 °C.
Set the annealing temperature for 1 min and 30 s at 54 °C.
Extension temperature set for 60 s at 72 °C.
Set final extension for 5 min at 72 °C.
Adjust 35 cycles for reaction at the end.
**Profile for *LukD* amplification**
Set 95 °C initial temperature for 320 s.
Set the time for denaturation to 60 s at 95 °C.
Adjust the annealing temperature to 58 °C for 90 s.
Allow 60 s for the extension at 72 °C.
Adjust to 600 s for the final extension at 72 °C temperature.
Then adjust 35 cycles for the PCR reaction.
Adjust the storage temperature to 4 °C at the end.
**Profile for *spa* amplification**
Set 95 °C initial temperature for 300 s.
Set the time for denaturation to 60 s at 95 °C.
Adjust the annealing temperature to 56 °C for 90 s.
Allow 60 s for the extension at 72 °C.
Adjust to 600 s for the final extension at 72 °C temperature.
Then adjust 35 cycles for the PCR reaction.
Adjust the storage temperature to 4 °C at the end.
**Profile for *FmhA* amplification**
Set 95 °C initial temperature for 320 s.
Set the time for denaturation to 60 s at 95 °C.
Adjust the annealing temperature to 54 °C for 90 s.
Allow 90 s for the extension at 72 °C.
Adjust to 300 s for the final extension at 72 °C temperature.
Then adjust 38 cycles for the PCR reaction.
Adjust the storage temperature to 4 °C at the end.
**Profile for *delta hemolysin* gene amplification**
Set 95 °C initial temperature for 300 s.
Set the time for denaturation to 60 s at 95 °C.
Adjust the annealing temperature to 60 °C for 90 s.
Allow 60 s for the extension at 72 °C.
Adjust to 300 s for the final extension at 72 °C temperature.
Then adjust 38 cycles for the PCR reaction.
Adjust the storage temperature to 4 °C at the end.

**Table 3:** List of B-cell epitopes along with their antigenicity and allergenicity.

Serial. No.	Epitope	Antigenicity
1	NADQRNGFIQSLKDDPSQSAN	0.591
2	AQKLNDSQAPKADAQQNNFN	0.987
3	KDQQSA	1.462
4	YEILNMPNLNEAQLTAEAAA	0.576
5	KEAAAKEAAAKMDYTNQSL	0.846
6	ENLRNADGDPRVPSS	0.964

**Table 4:** List of MHC-I epitopes along with their antigenicity and allergenicity.

Alleles	Epitopes	Antigenicity
HLA-A*11:01	QSLVAFFFK	0.624
HLA-A*68:01		
HLA-A*31:01		
HLA-A*02:06	KQNCLYVLV	0.636
HLA-A*02:03		
HLA-A*02:06	AQLTAEAAA	0.854
HLA-B*53:01	FPSHLCDLVI	1.017
HLA-A*11:01	NQSLVAFFFK	0.636
HLA-A*68:01		
HLA-A*02:01	SLVAFFFKSL	0.501
HLA-A*02:06		
HLA-A*30:01	KSIYFPIFL	2.125
HLA-B*58:01		
HLA-A*02:01	YVLVDPYLI	2.058

**Table 5:** List of MHC- II epitopes along with their antigenicity and allergenicity.

Alleles	Epitopes	Antigenicity
HLA-DRB1*07:01	VILKHAKSIYFPIFL	0.981
HLA-DRB1*15:01		
HLA-DRB1*01:01		
HLA-DRB1*13:02		
HLA-DRB1*12:01		
HLA-DPA1*01:03/DPB1*02:01, HLA-DPA1*01:03/DPB1*04:01, HLA-DRB1*09:01		
HLA-DRB1*08:02		
HLA-DRB5*01:01		
HLA-DRB1*15:01	LKIKQLINYFPSHLC	0.632
HLA-DRB1*08:02		
HLA-DRB1*13:02		
HLA-DRB1*12:01		
HLA-DRB1*07:01		
HLA-DRB1*01:01		
HLA-DRB1*04:05		
HLA-DRB1*09:01		
HLA-DRB1*04:01		
HLA-DRB1*12:01	IKIKILKIKQLINYF	0.591
HLA-DRB4*01:01		
HLA-DRB1*15:01		
HLA-DRB1*11:01		
HLA-DRB1*01:01		
HLA-DPA1*03:01/DPB1*04:02 HLA-DRB1*08:02		
HLA-DPA1*03:01/DPB1*04:02, HLA-DRB4*01:01	ITPSLIKIKILKIKQ	0.834
HLA-DRB1*11:01		
HLA-DRB1*12:01		
HLA-DRB1*15:01		
HLA-DRB1*01:01		
HLA-DQA1*01:01/DQB1*05:01		
NCLYVLVDPYLIENL HLA-DQA1*01:01/DQB1*05:01, HLA-DRB3*01:01		
HLA-DRB1*01:01		
HLA-DRB1*07:01		
HLA-DRB1*04:05		
HLA-DPA1*01:03/DPB1*02:01, HLA-DPA1*03:01/DPB1*04:02, HLA-DRB1*15:01		
HLA-DRB1*01:01	NCLYVLVDPYLIENL	1.003

**Table 6:** Physiochemical Analysis of Vaccine Construct.

Property	Measurement	Indication
Total number of amino acids	405	Appropriate
Molecular weight	43,773.14 kDa	Appropriate
Formula	C_1988_H_3093_N_533_O_566_S_8_	-
Theoretical pI	9.44	Basic
Total number of positively charged residues (Arg + Lys)	28	-
Total number of negatively charged residues (Asp + Glu)	45	-
Total number of atoms	6188	-
Instability index (II)	32.82	Stable
Aliphatic index	85.70	Thermostable
Grand Average of Hydropathicity (GRAVY)	−0.195	Hydrophilic
Antigenicity VaxiJen	0.96	Antigenic
Antigenicity AntigenPro	0.731	Antigenic
Allergenicity	Non-allergenic	Non-allergenic
Toxicity	Nontoxic	Nontoxic

## Data Availability

Not applicable.
